# Evolving Nutritional Therapy for Diabetes Mellitus

**DOI:** 10.3390/nu12020423

**Published:** 2020-02-06

**Authors:** Matti Uusitupa, Ursula Schwab

**Affiliations:** 1 Institute of Public Health and Clinical Nutrition, University of Eastern Finland, P.O. Box 1627, 7211 Kuopio, Finland; ursula.schwab@uef.fi; 2 Department of Medicine, Endocrinology and Clinical Nutrition, Kuopio University Hospital, 70210 Kuopio, Finland

One of the special issues in *Nutrients* in 2020 focuses on the nutritional therapy for diabetes mellitus. Altogether, nine articles were published in the special issue of the Journal dealing with different aspects of the quality of diet in the treatment or prevention of diabetes by dietary patterns or special nutrients. The topic is of utmost importance since the prevalence of diabetes is still increasing worldwide. In particular, this concerns type 2 diabetes that is closely related to an increasing prevalence of overweight and obesity, sedentary lifestyles and unhealthy dietary choices [1,2]. The American Diabetes Association (ADA) publishes on a yearly basis updated dietary recommendations for the nutritional therapy of diabetes [3], but the latest European guidelines were published in 2004 [4]. Furthermore, there are many national guidelines that are more or less in line with the above-mentioned guidelines, and a consensus paper by ADA and the European Association for the Study of Diabetes (EASD) also includes dietary guidelines [5]. The new European guidelines are aiming to be published in 2020, and some of the papers published in the special issue of *Nutrients* serve as a background for the coming new recommendations.

When considering the dietary guidelines for diabetes, it is of importance to recognize that there are big differences in the management of type 1 and type 2 diabetes. Furthermore, in particular, type 2 diabetes is not a single disease regarding its phenotype or genotype [6,7]. However, current dietary recommendations regarding diet therapy for diabetes make no big difference between type 1 and type 2 diabetes. Whereas type 1 diabetes is an autoimmune disease leading to severe or complete insulin secretion defect without solid evidence of any dietary aetiologies, type 2 diabetes is closely related to several lifestyle and dietary factors leading to overweight and obesity with concomitant insulin resistance in the liver, muscle, and adipose tissue [8]. Genetically, type 2 diabetes is a heterogenic disease; some 400 genetic variants have been identified to increase the risk of type 2 diabetes. Most of them are related to insulin secretion defects, but genetic variants related to obesity and insulin resistance also seem to play a role in the development of type 2 diabetes [7]. Current evidence strongly suggests that, in most cases, lifestyle changes along with weight reduction seem to overcome the genetic risk of the disease in people with prediabetes or recent type 2 diabetes [9,10].

The ultimate goal of the management of diabetes is to control hyperglycemia on a daily basis and prevention of acute and long-term complications of diabetes, including macro- and microvascular diseases and other long-term disabilities related to diabetes. In particular, cardiovascular diseases, severe retinal diseases, and chronic renal disease among diabetic patients are major challenges for health care systems worldwide. With regard to type 1 diabetes, insulin therapy is nowadays often based on continuous glucose monitoring, at least in high-income countries. The development of glucose monitoring techniques and multiple insulin injections or insulin pumps have resulted in better long-term glucose control and improved prognosis of patients with type 1 diabetes. Still, healthy dietary patterns are one of the cornerstones of diet therapy for patients with type 1 diabetes. There is no particular need to make any special changes regarding protein intake except in patients with renal disease. As for carbohydrates, high quality of carbohydrate sources, like whole grain products, fruit, vegetables, and pulses, are preferable, and the total carbohydrate intake may be in line with the recommendations for the general population [11,12,13]. Counting carbohydrates is important and helps to adjust insulin therapy accordingly [3]. Similarly, physical activity should be taken into account when it comes to carbohydrate intake [3,14]. Sugar containing products should be used in moderation (sugar less than 10 E%) due to their potential effects on glucose control, and in long-run, body weight control, as well. New sweeteners may offer some additional benefits [15]. Regarding the intake of fat in diabetic patients, both the quality and quantity of fat are in line with the general recommendations regardless of the type of diabetes [4].

Because both main types of diabetes increase the risk of atherosclerotic vascular diseases, the dietary guidelines for nutritional therapy for diabetes emphasize the use of unsaturated fatty acids instead of saturated (<7–10 E%) and trans-unsaturated fatty acids, which should be used as little as possible.

The attitude to high protein diets is controversial, as the current ADA’s recommendations [3] and that of the Diabetes and Nutrition Study Group (DNSG) for EASD are aiming to present [16]. This controversy originates from the fact that there are no real long-term intervention trials on high protein diets in people with diabetes [16]. Furthermore, ADA’s recommendations also include some reservations with regard to high protein diets [3]. A recent meta-analysis published in *Nutrients* concludes that the safety of high protein diets has been documented for one year only [16]. As said above, the quality or quantity of dietary protein does not seem to play any role in the pathogenesis of type 1 diabetes, whereas, regarding type 2 diabetes, higher intakes of red meat and processed meat products have been consistently associated with the increased risk of type 2 diabetes [12] and other chronic non-communicable diseases [13]. This should arouse concern when considering any dietary guidelines. It is of note that the current ADA’s recommendations make no difference with regard to animal vs. vegetable protein sources. A high protein diet may even hamper the goals of dietary guidelines, e.g., increasing the intake of whole-grain products, fruit and vegetables, and dietary fiber that are currently low in many westernized diets. One argument not to recommend high protein diets in the longer-term is that this kind of a diet may cause concern when adjusting dietary patterns to a lower protein intake when necessary, e.g., regarding the development of renal diseases [3]. Furthermore, in the future, ecologic issues should be considered with regard to dietary recommendations [17]. Finally, all the long-term prevention trials of type 2 diabetes have applied diets emphasizing healthy dietary patterns with normal protein intake [9]. The primary goal of the prevention of type 2 diabetes is permanent weight reduction that can be achieved by healthy dietary patterns and increased physical activity. So far, no long-term prevention studies with a high protein diet exist.

The DiRECT study results have shown that substantial weight reduction may also result in the remission of recent type 2 diabetes [10] along with diminished fat content in the liver and pancreas, reemphasizing the importance of active management of obesity, both in prediabetes and type 2 diabetes, during its early years.

Personalized diets are emerging, but a priori current dietary recommendations do not take into account the putative genetic or phenotypic heterogeneity of diabetes. Furthermore, the role of gut microbiota may, in the future, modify dietary recommendations. What we already may recommend with reasonable evidence is polyunsaturated fatty acids instead of saturated ones, not only for the prevention of atherosclerosis but also for the prevention and treatment of fatty liver [18,19], common in people with type 2 diabetes, but the response could be different according to genetic background [20]. Increasing the intake of whole grains and dietary fiber due to their potential health benefits with regard to gut microbiota and the prevention of non-communicable diseases makes sense without any doubt, but the response may be dependent, e.g., on the quality of gut microbiota. Still, based on the current scientific evidence, individually tailored diets for patients with diabetes are far from clinical practice.

To conclude, new findings based on nutritional science help us to update the dietary recommendations for patients with diabetes. There is not enough data for personalized dietary guidelines so far except for some monogenic types of diabetes [3]. Besides basic research, well-controlled dietary interventions with hard end-points are still needed, in particular with regard to the role of the gut and the putative role of the variety of phenotypes and genotypes of type 2 diabetes. Fortunately, in principle, healthy dietary patterns fit well for most people with diabetes [21].
